# Prenatal tests in Brazil: prevalence and associated factors according to the Brazilian National Health Survey

**DOI:** 10.1590/1980-220X-REEUSP-2024-0154en

**Published:** 2025-01-27

**Authors:** Francisca Maria da Silva Freitas, Rita Da Graça Carvalhal Frazão Correia, Camila Biazus-Dalcin, Herla Maria Furtado Jorge, Priscila de Souza Aquino, Bruno Luciano Carneiro Alves de Oliveira

**Affiliations:** 1 Universidade Federal do Maranhão, Departamento de Enfermagem, São Luís, MA, Brazil.; 2 University of Dundee, School of Health Sciences, Dundee, Scotland.; 3 Universidade Federal do Piauí, Departamento de Enfermagem, Teresina, PI, Brazil.; 4 Universidade Federal do Ceará, Departamento de Enfermagem, Fortaleza, CE, Brazil.; 5 Universidade Federal do Maranhão, Departamento de Medicina I, São Luís, MA, Brazil.

**Keywords:** Prenatal Care, Laboratory Test, Health Services Accessibility, Health Surveys, Nursing, Atención Prenatal, Prueba de Laboratorio, Accesibilidad a los Servicios de Salud, Encuestas Epidemiológicas, Enfermería

## Abstract

**Objective:**

To analyze the prevalence of prenatal tests of pregnant women and factors associated with variation in this prevalence in the years of the Brazilian National Health Survey 2013 and 2019.

**Method:**

A cross-sectional study, carried out with women who underwent prenatal care, interviewed in the Brazilian National Health Survey 2013 (n = 1,851) and 2019 (n = 2,729).

**Results:**

The most prevalent tests were urine and blood, and the least prevalent were syphilis and HIV. During the period, the number of tests for syphilis (15.2; 95% CI: 11.0; 22.0) and HIV (4.3; 95% CI: 4.3; 8.0) increased, but the number of tests for the others decreased. The prevalence of tests for the four tests increased and reached 69.9% (95% CI: 67.0; 72.8) in 2019 compared to 60% (95% CI: 56.1; 63.9) in 2013.

**Conclusion:**

There was a greater number of prenatal tests performed, specifically for syphilis and HIV, rather than a reduction in the number of blood and urine tests. Despite the increase in access to all tests for the most vulnerable groups and locations in the country, prevalence in these groups is still low.

## RESUMEN

**Objetivo:**

Analizar la prevalencia de exámenes prenatales en mujeres embarazadas y los factores asociados a la variación de esta prevalencia en los años de la Encuesta Nacional de Salud 2013 y 2019.

**Método:**

Estudio transversal, realizado con mujeres que realizaron control prenatal, entrevistadas en la Encuesta Nacional de Salud de 2013 (n = 1.851) y 2019 (n = 2.729).

**Resultados:**

Las pruebas de mayor prevalencia fueron orina y sangre, y las menos prevalentes fueron sífilis y VIH. Durante el período, el número de pruebas de sífilis (15,2; IC 95%: 11,0; 22,0) y VIH (4,3; IC 95%: 4,3; 8,0) aumentó, pero el de los demás disminuyó. La prevalencia de la realización de los cuatro exámenes aumentó y alcanzó el 69,9% (IC95%: 67,0; 72,8) en 2019, frente al 60% (IC95%: 56,1; 63,9) en 2013.

**Conclusión:**

Hubo mayor número de exámenes prenatales realizados, específicamente para sífilis y VIH, en detrimento de una reducción de los exámenes de sangre y orina. A pesar del crecimiento en el acceso a todas las pruebas para los grupos y ubicaciones más vulnerables del país, la prevalencia en estos grupos aún es baja.

## INTRODUCTION

Prenatal care is essential for maternal and neonatal health, as it allows for early identification and timely treatment of problems that may occur during pregnancy and postpartum, in addition to reducing maternal, fetal and infant morbidity and mortality^(1)^. However, millions of pregnant women around the world still do not receive them adequately. The 3^rd^ Sustainable Development Goal (SDG) of the United Nations establishes the target of reducing maternal and infant morbidity and mortality, which requires global efforts to achieve universal access to healthcare services and supplies, such as access to prenatal care with tests^(2,3)^.

Despite being a fundamental part of achieving global goals, this access still represents a problem of great magnitude^(4)^. In the United States of America (USA), disparities persist and are significant. Women born in the USA have higher rates of prenatal care compared to immigrants^(5)^. In England, quality of care is unsatisfactory due to inequality in access and late referrals. Furthermore, prenatal tests are not yet uniform across population groups and locations in the country^(6)^.

A study of Brazilian pregnant women revealed a prevalence of access to prenatal tests of less than 37.3%^(7)^. Another study conducted with 408 postpartum women in three reference maternity hospitals in the state of Paraná, in southern Brazil, revealed a prevalence of less than 50% during pregnancy^(8)^. The reasons for this are attributed to the Social Determinants of Health, as individual characteristics (lower education, income, age, absence of a partner) and contextual characteristics (region of residence and type of services used) of the mother lead to fewer tests and prenatal coverage^(9,10,11)^.

The Brazilian Ministry of Health^(12)^ recommends routine laboratory tests and rapid tests during prenatal care, including blood tests to detect syphilis, HIV, hepatitis B and C, serology for toxoplasmosis, gestational diabetes, anemia and blood typing with Rh factor as well as urine tests. These can be performed in primary healthcare.

Despite this, in Brazil, studies on prevalence and factors associated with prenatal tests are still less frequent in the academic literature than those on prenatal care adequacy based on the analysis of the number of consultations and the period of initiation. The ordering of priorities of the Ministry of Health, the political organization in the Healthcare Network and the Stork Network strategy depend on this knowledge as a way of assessing and monitoring the implementation of public policies, organization of healthcare services and management of care for pregnant women and their newborns^(8,9,10,11,12,13)^.

Therefore, analyses on the prevalence of prenatal tests and factors associated with the variation in this prevalence in Brazil are still scarce, which contributes to maintaining the lack of knowledge about the health problems that affect pregnant women that involve the risk of vertical transmission during pregnancy or labor.

Therefore, this study sought to analyze the prevalence of access to prenatal tests for pregnant women and factors associated with the variation in this prevalence in Brazil.

## METHOD

### Study Design

This is a cross-sectional study. The article was written in accordance with STrengthening the Reporting of OBservational studies in Epidemiology (STROBE) guidelines^(14)^.

### Place

The data came from the Brazilian National Health Survey (In Portuguese, *Pesquisa Nacional de Saúde* - PNS) 2013 and 2019, which is a nationwide population-based household survey. It collects valid and representative information about the Brazilian population about their living conditions^(15,16,17)^. PNS databases were obtained from the FIOCRUZ website^(18)^. All stages of variable coding and analysis were carried out by the authors of this work.

### Population and Selection Criteria

The target population in 2013 was individuals aged ≥18 years and, in 2019, ≥15 years, living in permanent private households in Brazil. The questionnaires cover households and all their residents^(16)^. The sampling used was probabilistic by cluster in three selection stages, with stratification by selected areas^(17)^. Further details can be obtained in PNS publications^(15,17)^.

### Sample Definition

In this study, only women aged 18 to 49 who were pregnant in the two years prior to the date of PNS data collection and received prenatal care were considered. In 2013, 1,851 women comprised the sample, and in 2019, another 2,729.

### Data Analysis and Treatment

In PNS, women who were pregnant in the two years prior to the interview date were asked about whether they had had a blood test (without considering the pregnancy test), urine test (without considering the pregnancy test), syphilis and HIV/AIDS tests, and the location where they received prenatal care (only public, only private, and public and private). For these tests, the possible answers were “yes”, “no”, “do not know/do not remember” or “unknown”. For each of these, the answers were aggregated: yes vs. the others. The four tests were also aggregated to verify whether they had been performed together (yes, no). For both years of PNS, the prevalence and their 95% Confidence Intervals (95%CI) of socioeconomic and demographic variables and location of prenatal care were estimated. Differences in the distribution of frequencies of variables were verified according to the year, and were considered statistically significant at the 5% level in the absence of overlapping 95%CI. Pearson’s chi-square test was used to confirm differences between the two PNS.

The prevalence and respective 95% CI of individual and combined tests were estimated for each year of PNS. The change in prevalence in the two years of PNS was presented through the absolute difference. The magnitude of this variation in the period was computed with a Generalized Linear Model (GLM), using the Gaussian distribution. To perform data analysis in the present study, the data from the two surveys were aggregated into a single database. Using the weight of the resident selected with calibration, absolute changes from 2013 to 2019 were calculated according to sociodemographic characteristics, parity, and prenatal care locations. To calculate the change in prevalence from 2013 to 2019, the effect of the year on the outcome was modeled according to the cluster variable. The reported percentage prevalence rate was calculated as the exponential of the coefficient minus one and multiplied by 100. Finally, Poisson regression models, robust variance, unadjusted and adjusted for sociodemographic, parity and prenatal location variables were performed to test the association of the year of PNS (2019 vs 2013) with access to prenatal tests. All analyses were performed using RStudio version 2023.6.1.524 (R Foundation for Statistical Computing, Boston, United States of America).

### Ethical Aspects

The data from the PNS 2013 and 2019 are in the public domain and can be used in accordance with the research of interest. The research was approved by the Brazilian National Research Ethics Commission/National Health Council (Process 328,159, issued in July 2013; Process 3,529,376, issued in August 2019). All interviewees signed the Informed Consent Form^(19)^.

## RESULTS

The sample was composed of data from 4,580 pregnant women interviewed. Table 1 shows data related to sociodemographic characteristics, parity and location of prenatal care of pregnant women in the 2013 and 2019 PNS.

**Table 1:** Sociodemographic characteristics, parity and location of prenatal care of pregnant women interviewed in the Brazilian National Health Survey – Brazil, 2013 and 2019.

Characteristics	2013 (n = 1,851)			2019 (n = 2,729)			p-valor^1^
	n	%	95%CI	n	%	95%CI	
**Total**	1801	97.3	96.4; 98.3	2680	98.2	97.6; 98.9	
Age group (in years)							
18 to 29	1135	61.3	57.5; 65.0	1441	52.8	49.5; 56.0	***0.001***
30 to 39	652	35.2	31.6; 38.3	1133	41.5	38.9; 44.8	
40 to 49	65	3.5	2.2; 4.3	156	5.7	4.7; 7.2	
Color/race						
Brown	926	50.0	46.2; 53.8	1397	51.2	48.0; 54.5	0.20
White	739	39.9	36.1; 43.7	980	35.9	32.8; 39.1	
Black	163	8.8	6.7; 10.9	308	11.3	9.2; 13.5	
Others	24	1.3	0.6; 2.0	41	1.5	1.0; 2.1	
Marital status								
No spouse	1048	56.6	52.9; 60.4	1586	58.1	55.0; 61.2
With spouse	803	43.4	39.6; 47.1	1143	41.9	38.8; 45.0	0.56
Parity							
One birth	731	39.5	35.8; 43.3	1064	39	36.1; 42.0	
Two to four births	1024	55.3	51.5; 59.2	1536	56.3	53.3; 59.3	0.86
Five or more births	94	5.1	3.9; 6.4	128	4.7	3.5; 5.9	
Participation in religious activities							
Yes	857	46.3	42.5; 50.1	1318	48.3	45.0; 51.5	0.45
No	994	53.7	49.9; 57.5	1411	51.7	48.5; 55.0	
Health insurance							
Yes	515	27.8	24.0; 31.6	742	27.2	24.0; 30.3	0.82
No	1336	72.2	68.4; 76.0	1987	72.8	69.7; 76.0	
Education							
Up to incomplete elementary school or equivalent	426	23.0	19.9; 26.0	491	18.0	15.7; 20.2	***0.001***
Incomplete high school or equivalent	446	24.1	20.8; 27.3	519	19.0	16.5; 21.5	
Incomplete higher education or equivalent	761	41.1	37.1; 45.0	1190	43.6	40.4; 46.8	
Complete higher education	220	11.9	9.5; 14.3	532	19.5	16.7; 22.2	
Household income per capita							
Up to ½ minimum wage	796	43.0	39.1; 46.8	1214	44.5	41.4; 47.7	0.95
½ to 1 minimum wage	546	29.5	26.2; 32.9	780	28.6	25.4; 31.7	
1 to 2 minimum wages	324	17.5	14.2; 20.8	445	16.3	13.6; 19.1	
2 to 3 minimum wages	76	4.1	2.5; 5.6	117	4.3	2.8; 5.9	
More than 3 minimum wages	109	5.9	4.2; 7.6	169	6.2	4.8; 7.7	
Macroregion of the country							
Southeast	707	38.2	34.3; 42.2	1051	38.5	35.4; 41.6	
Northeast	533	28.8	25.5; 32.1	778	28.5	26.2; 30.7	
South	270	14.6	11.9; 17.4	355	13.0	11.5; 14.6	0.81
North	185	10.0	8.5; 11.6	308	11.3	10.1; 12.6	
Midwest	154	8.3	7.0; 9.6	237	8.7	7.4; 9.9
Type of city							
Inland	1068	57.7	54.2; 61.2	1676	61.4	58.8; 64.0	0.18
Capital or Metropolitan Region	783	42.3	38.8; 45.8	1053	38.6	36.0; 41.2	
Place where they received their prenatal care							
Public only	1283	69.3	65.5; 73.0	1771	64.9	61.6; 68.2	0.06
Private only	500	27.0	23.3; 30.6	794	29.1	26.0; 32.2	
Public and private	70	3.8	2.5; 5.1	164	6.0	4.7; 7.3	

Note: 95%CI – 95% Confidence Interval

^1^Pearson’s chi-square test.

Source: PNS 2013 and 2019.

The proportion of pregnant women who reported having received prenatal care in Brazil increased by 0.9% over the years. Although the age group of 18 to 29 years is the most prevalent, a reduction was observed in this group and an increase in the other age groups. The predominant pregnant women were brown, without a partner, with two to four births, without religious activities, without health insurance, with an income of up to ½ minimum wage, living in the southeast, in the countryside and who received prenatal care only in public services. Over the years, a significant increase was observed in the number of pregnant women with higher education, incomplete higher education and complete higher education, with a consequent reduction in the lowest levels of education. Table 2 shows the data regarding the prevalence of tests performed by pregnant women.

**Table 2:** Prevalence and change in the prevalence of prenatal tests among pregnant women interviewed in the Brazilian National Health Survey – Brazil, 2013 and 2019.

Tests	2013 (n = 1,851)			2019 (n = 2,729)			Absolute change* (2019 vs 2013)		
	n	%	95%CI	n	%	95%CI	%	95%CI
Urine	1816	98.1	97.2; 98.9	2541	93.1	91.2; 95.0	–5.0	–6.8; –2.8
Blood	1801	97.3	96.2; 98.4	2573	94.3	92.6; 96.1	–3.0	–4.9; –0.9
HIV	1575	85.1	82.3; 87.8	2440	89.4	87.9; 91.1	4.3	1.0; 8.0
Syphilis	1196	64.6	60.8; 68.5	2178	79.8	77.4; 82.2	15.2	11.0; 22.0
**Total**	1111	60.0	56.1; 63.9	1908	69.9	67.0; 72.8	9.9	5.0; 16.0

Note: 95%CI – 95% Confidence Interval

*GLM.

Source: PNS 2013 and 2019.

**Figure 1:**
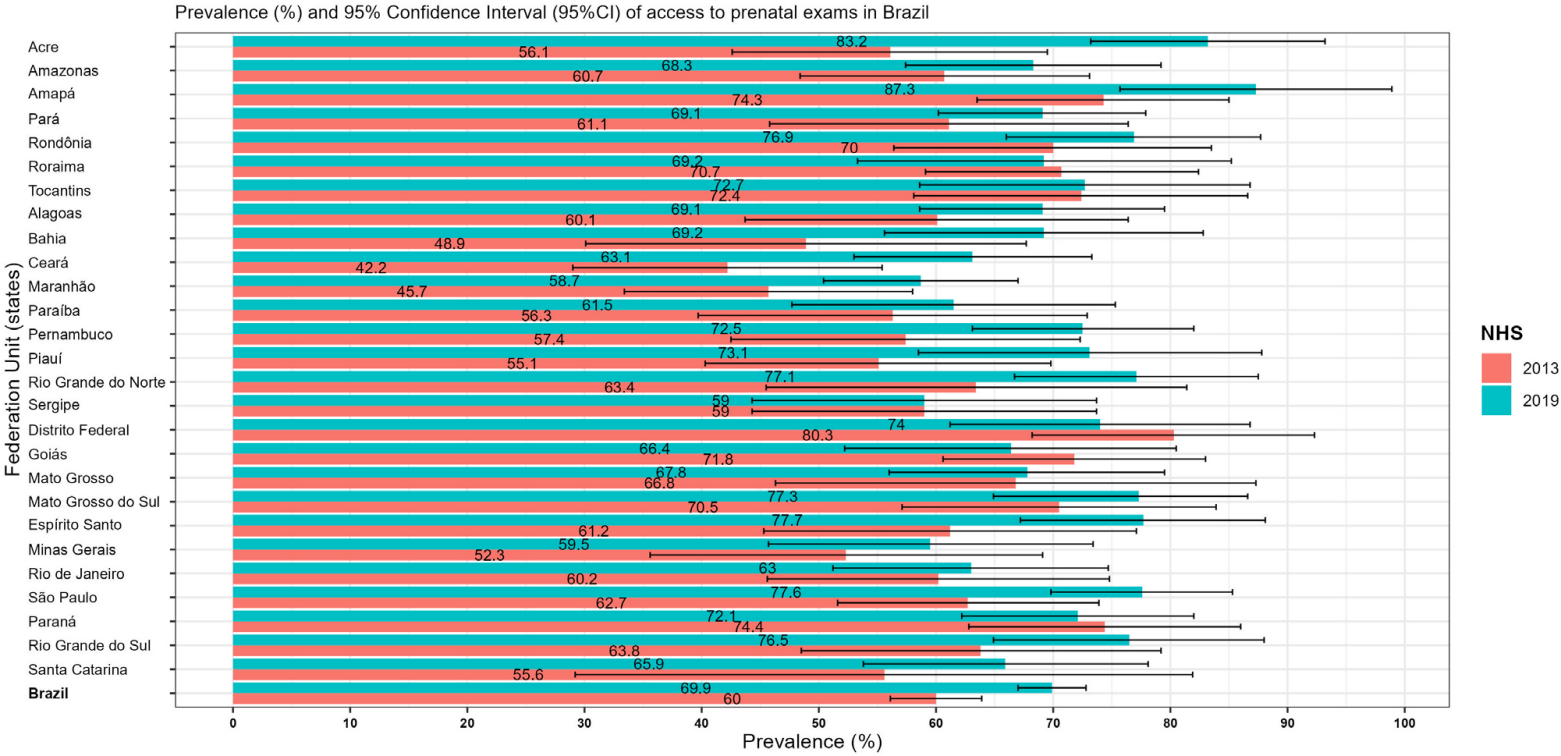
Prevalence (%) and 95% Confidence Intervals of the performance of the set of prenatal tests of pregnant women interviewed in the Brazilian National Health Survey, Brazil, 2013 and 2019.

Source: Brazil, NHS 2013 and 2019

Notes: Between years, all exams p-value < 0.01.

As can be seen in Table 2, there was a significant reduction in the number of blood and urine tests performed by the pregnant women interviewed and a significant increase in the number of tests for syphilis, HIV and the total number of tests performed in the years of PNS. Figure 1 shows the data regarding the performance of the set of prenatal tests by the Federation Units.

Figure 1 shows that in 2013 there was a significant difference in access to prenatal care: Ceará had the lowest prevalence when compared to Amapá, Rondônia, Roraima, Tocantins, Goiás, Mato Grosso do Sul and Paraná. In 2019, the lowest estimates were from the states of Maranhão, Ceará and Minas Gerais. There was a significant difference between Maranhão, which had the lowest prevalence, and Acre, Amapá and São Paulo. In the years, only the state of Acre showed a statistically significant increase, as did Brazil in general, indicating an increase in access to the tests investigated.

Data relating to prenatal tests according to sociodemographic characteristics, parity and location of prenatal care for pregnant women are shown in Table 3.

**Table 3:** Prevalence and change in the prevalence of prenatal tests according to sociodemographic characteristics, parity and location of prenatal care among pregnant women interviewed in the Brazilian National Health Survey – Brazil, 2013 and 2019.

Characteristics	2013 (n = 1,851)		2019 (n = 2,729)		Absolute change* (2019 vs 2013)	
	%	95%CI	%	95%CI	%	95%CI
**Total**	60.0	56.1; 63.9	69.9	67.0; 72.8	9.9	5.0; 16.0
Age group (in years)						
18 to 29	55.1	49.9; 60.3	67.1	63.0; 71.2	12.1	5.0; 21.0
30 to 39	67.7	61.7; 73.8	73.3	69.0; 77.5	6.0	–1.6; 14.0
40 to 49	67.5	52.7; 82.3	71.2	59.6; 82.8	3.7	–13.9; 25.0
Color/race						
White	62.5	55.9; 69.1	68.5	63.1; 74.0	6.0	–2.4; 16.0
Brown	58.2	53.0; 63.4	71.6	68.0; 75.1	14.0	7.0; 22.0
Black	58.2	45.5; 71.0	68.7	60.5; 76.8	10.5	–4.4; 29.0
Others	62.2	34.3; 90.2	55.9	37.7; 74.1	–6.3	–32.2; 30.0
Marital status						
With spouse	62.9	57.1; 68.8	70.6	66.2; 75.0	8.0	1.0; 16.0
Without spouse	57.7	52.6; 62.9	69.4	65.5; 73.2	11.7	5.0; 20.0
Birth						
One birth	65.0	58.1; 70.9	69.2	64.2; 74.1	5.0	–3.2; 13.0
Two to four births	56.8	51.5; 62.0	70.7	67.1; 74.4	15.0	8.0; 22.0
Five or more births	60.1	48.5; 71.7	66.0	53.1; 79.0	6.0	–10.9; 26.0
Participates in religious activities						
Yes	62.4	56.4; 67.9	72.2	68.4; 76.4	9.8	3.0; 18.0
No	58.0	53.0; 62.9	67.8	63.8; 71.8	9.8	3.0; 18.0
Health insurance						
Yes	69.2	61.5; 76.8	75.7	70.0; 81.4	6.5	–2.9; 17.0
No	56.5	52.0; 61.0	67.7	64.4; 71.1	11.2	6.0; 18.0
Education						
Up to incomplete elementary school or equivalent	42.8	35.8; 49.9	57.8	51.2; 64.4	15.0	6.0; 27.0
Incomplete high school or equivalent	60.0	52.4; 67.7	64.9	58.0; 71.7	4.9	–5.0; 16.0
Incomplete higher education or equivalent	65.8	59.1; 72.4	74.1	69.8; 78.4	9.0	–1.0; 17.0
Complete higher education	73.0	62.8; 83.2	76.6	70.4; 82.9	4.0	–8.2; 17.0
Household income per capita						
Up to ½ minimum wage	51.0	45.4; 56.7	66.4	62.4; 70.5	17.0	9.0; 25.0
½ to 1 minimum wage	66.6	59.9; 73.4	70.2	64.3; 76.1	4.0	–5.4; 14.0
1 to 2 minimum wages	63.4	52.0; 74.8	79.0	72.1; 85.9	15.6	–2.0; 34.0
2 to 3 minimum wages	62.7	42.7; 82.8	70.5	57.2; 83.8	7.8	–15.0; 37.0
More than 3 minimum wages	79.8	68.0; 91.6	69.0	57.7; 80.4	–10.2	–23.4; 5.0
Macroregion of the country						
North	63.8	56.1; 71.6	71.5	66.3; 76.8	7.7	–1.2; 19.0
Northeast	51.5	44.9; 58.1	67.2	62.7; 71.7	17.0	8.0; 27.0
Midwest	72.0	64.6; 79.4	70.1	63.3; 76.9	–1.2	–11.3; 9.0
Southeast	60.1	52.4; 67.7	70.6	64.6; 76.5	10.5	–1.0; 22.0
South	67.1	57.2; 76.9	72.3	65.9; 78.8	5.0	–6.5; 19.0
Type of city						
Capital or Metropolitan Region	68.2	63.8; 72.5	73.0	69.4; 76.7	4.8	–0.9; 11.0
Inland	54.0	48.1; 60.0	67.9	63.8; 72.0	15.0	7.0; 24.0
Place where prenatal care was provided						
Only private	73.0	66.3; 79.7	72.2	66.5; 77.8	–0.8	–8.9; 8.0
Only public	54.5	49.8; 59.2	67.5	63.9; 71.0	14.0	7.0; 21.0
Public and private	72.4	66.0; 78.8	74.4	69.4; 79.3	2.0	–5.8; 1.0

Notes: 95%CI – 95% Confidence Interval

*GLM.

Source: PNS 2013 and 2019.

As can be seen in Table 3, the number of tests performed increased in the years of the research, with a statistically significant difference for younger pregnant women (18 to 29 years old), brown, without a partner, with two to four births, without health insurance, with lower education, lower income, living in the northeast, in the countryside and with prenatal care performed only in the public network.

**Figure 2:**
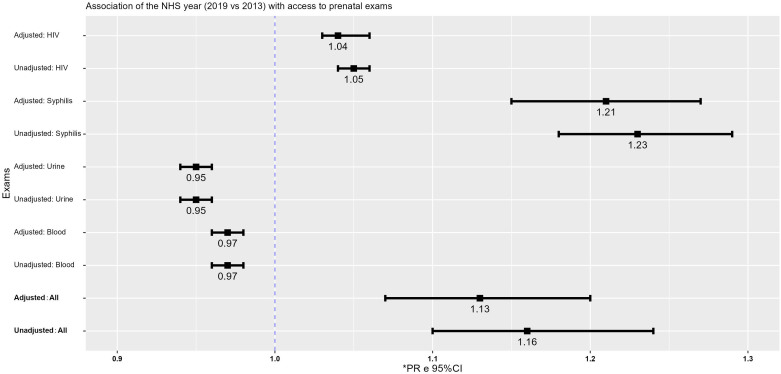
Unadjusted and adjusted association of prenatal tests of pregnant women with the year in which they were interviewed in the Brazilian National Health Survey, Brazil, 2013 and 2019.

Source: Brazil, NHS 2013 and 2019.

Figure 2 shows the unadjusted and adjusted association of the year of PNS with the simultaneous and specific performance of prenatal tests.

*Poisson regression with robust variance. Adjustment made based on the variables present in Table 1.

As can be seen in Figure 2, there was an improvement in simultaneous access to all tests. In 2019, compared to 2013, after adjusting for confounding factors, pregnant women had a 13% (PR = 1.13; 95% CI: 1.07–1.20) greater chance of undergoing the tests investigated. When broken down by test, adjusted analysis also indicated an improvement in access to syphilis and HIV tests, with greater chances: 21% (PR = 1.21; 95% CI: 1.15–1.27) and 4% (PR = 1.04; 95% CI: 1.03–1.06), respectively. On the other hand, pregnant women showed reduced access to blood tests (PR = 0.97; 95%CI: 0.96–0.98) and urine tests (PR = 0.95; 95%CI: 0.94–0.96).

## DISCUSSION

The results of this study showed an increase in the performance of all four prenatal tests, especially driven by syphilis and HIV tests, between 2013 and 2019 in Brazil. Although the prevalence is high, there was a reduction in access to blood and urine tests. The increase in the number of tests observed was for the most vulnerable groups and locations in the country. However, inequalities in access still persist, at individual and contextual levels, highlighting the need to intensify efforts to ensure more equitable access to prenatal laboratory tests throughout Brazil. It has been proven that timely, appropriate and equitable access to quality healthcare during pregnancy contributes to better health outcomes for women who give birth and for babies after birth^(20)^.

A comprehensive review of 68 systematic reviews from 20 different countries on equity in healthcare services highlighted some barriers to prenatal access, such as non-white patients, those with less education, lower income and those living in remote areas^(20)^.

In the present study, when comparing the 2013 and 2019 PNS studies, there was a significant increase in the number of tests performed for the group of pregnant women in the youngest age group of the study (18 to 29 years) and with the lowest level of education. In the USA, younger women, under 24 years of age and with the lowest level of education, confirmed their pregnancy later and received prenatal care later than they wanted, which could hinder adequate care and testing^(21)^. Another study carried out in 19 East African countries on the quality of prenatal care and associated factors among pregnant women showed similarities in these results, revealing that the quality of this care was higher in the age group of 20 to 34 years (69.44%) and with higher levels of education^(22)^. A study carried out in Indonesia with 8,239 working women showed greater access to prenatal consultations among those with partners, with a higher level of education, lower parity, higher income and health insurance, which is consistent with the data from the present study^(23)^.

In the Democratic Republic of São Tomé and Príncipe, of the pregnant women who had at least eight consultations, only 34% were able to undergo two hemoglobin tests and two urine tests, showing that, even with the appropriate number of follow-ups, pregnant women were unable to undergo the recommended screening during pregnancy. Considering that at least one test was performed, 95.5% were screened for HIV, 91.2% for syphilis, 60.1% for hepatitis, 75.5% for hemoglobin, 54.6% for blood glucose, and 70.3% for urine. The justification for not performing the test was the lack of reagent and the lack of financial support^(24)^. In another study in Lusaka, Zambia, on factors associated with HIV and syphilis screening among pregnant women at their first antenatal visit, only 29% of pregnant women were screened for syphilis, compared with 95% who were screened for HIV at their first antenatal visit^(25)^.

A study conducted in Fortaleza, in northeastern Brazil, which analyzed 560 prenatal consultations, showed that only 25.4% of pregnant women underwent all recommended tests adequately. This was the lowest adequacy found in prenatal care, rather than clinical-obstetric procedures, the number of consultations, and early access. The prevalence of adequacy was 30.9% for HIV, 36.2% for syphilis, and 34.4% for urine^(26)^.

One finding that deserves attention is the improvement in access for the most vulnerable groups. However, what is still observed is the marked inequality in access and quality of prenatal care with serious maternal and child repercussions related, in large part, to geographical difficulties and barriers to access to diagnostic and therapeutic services^(27)^. This reveals the urgent need to improve prenatal care for comprehensive care that responds to demands related to social and economic vulnerabilities, being essential for the prevention and early detection of maternal and fetal syndromes and pathologies through laboratory tests and, consequently, for the reduction of deaths^(28)^.

It should be noted that, since 2016, the Ministry of Health has declared a syphilis epidemic in Brazil. However, despite efforts to increase testing and treatment of the infection among pregnant women, congenital syphilis rates in the country remain high^(29)^. It is evident that the relationship between congenital syphilis and socioeconomic vulnerability factors. These determinants not only affect access to essential prenatal care services, but also the continuity and quality of such preventive measures^(30)^. Furthermore, it is noteworthy that knowledge about the partner’s syphilis test is essential to interrupt the chain of transmission, but this data is difficult to analyze, since in 2019 (21.9%) and in 2013 (99.6%) “not applicable” was recorded, with women without a partner.

There are major challenges to improving the provision of prenatal care and access to tests. There is an urgent need for health policies that contribute to access to prenatal laboratory tests for vulnerable populations so that health inequalities and their effects on maternal and infant mortality rates in the country can be reduced. This action is part of the SDG targets for 2030, and to achieve this, it requires the qualification of these services^(31)^.

### Study Limitations and Contributions

Despite these results, there are some limitations. Data from epidemiological surveys in which pregnant women self-reported information about tests may present recall and information bias. There were substantial changes in PNS regarding the way the question about tests was asked (in 2013: During prenatal care, did you have a blood test? And in 2019: During this pregnancy, did you have any blood test, other than the pregnancy test?). The different approach to the same test may have influenced the understanding and information processed during the interview, interfering with pregnant women’s responses. Another important point is related to the non-specification of other tests, in addition to HIV and syphilis, and the impossibility of distinguishing the type of blood and urine test performed (whether fasting blood glucose, complete blood count, serology).

However, this is a national survey of pregnant women from all over Brazil. The results indicate the importance of investigating access to routine prenatal tests. It can contribute to the reformulation of strategies that improve the quality of prenatal care in the country, in addition to contributing to the knowledge of healthcare professionals for assertive decision-making regarding the care of pregnant women, given the epidemiological profile of vulnerability presented.

## CONCLUSION

This study showed an increase in the performance of all four prenatal tests and also specifically for syphilis and HIV tests from 2013 to 2019 in Brazil. However, there was a reduction in blood and urine tests. This increase was for the most vulnerable groups and locations in the country, such as younger pregnant women (18 to 29 years old), brown skinned women, women without a partner, women with two to four births, women without health insurance, women with lower education levels, lower incomes, women living in the Northeast region, in the countryside, and women with prenatal care provided only in the public health system. However, the prevalence is still low, and there are still inequalities in access to tests in Brazil.

Despite the importance of this topic, published national studies have not yet analyzed the prenatal tests recommended in the country. Thus, this study indicates a path for a research agenda on the importance of investigating access to prenatal tests, which may contribute to the reformulation of intervention strategies aimed at improving care indicators for pregnant women using the Brazilian Health System.

Furthermore, it contributes to producing knowledge in the obstetric area within the scope of primary healthcare, favoring greater targeting of actions towards vulnerable pregnant women, as well as helping in the evaluation of the Brazilian reality, favoring comparison with other countries.

## References

[1] Mohseni M, Mousavi Isfahani H, Moosavi A, Mohammadian ED, Mirmohammadi F, Ghazanfari F (2023). Health system-related barriers to prenatal care management in
low-and middle-income countries: a systematic review of the qualitative
literature.. Prim Health Care Res Dev..

[2] Nações Unidas no Brasil. (2024). Objetivos de Desenvolvimento Sustentável (ODS) [Internet]..

[3] Organização das Nações Unidas. (2022). UNFPA: Brasil segue com índices elevados de gravidez na
adolescência [Internet].. https://brasil.un.org/pt-br/199938-unfpa-brasil-segue-com-%C3%ADndices-elevados-de-gravidez-na-adolesc%C3%AAncia.

[4] World Health Organization. (2016). WHO recommendations on antenatal care for a positive pregnancy
experience [Internet].. https://www.who.int/publications/i/item/9789241549912.

[5] Janevic T, Weber E, Howell FM, Steelman M, Krishnamoorthi M, Fox A. (2022). Analysis of state Medicaid expansion and access to timely
prenatal care among women who were immigrant vs US born.. JAMA Netw Open..

[6] McDonald H, Moren C, Scarlett J. (2020). Health inequalities in timely antenatal care: audit of pre-and
post-referral delays in antenatal bookings in London
2015–16.. J Public Health..

[7] Luz LA, Aquino R, Medina MG. (2018). Avaliação da qualidade da Atenção Pré-Natal no
Brasil.. Saúde Debate..

[8] Fabri ER, Canônico SB, Silva RMM, Ferreira H, Zilly A, Contiero AP. (2023). Prevalência e fatores associados à realização de exames
pré-natais na pandemia de COVID-19: um estudo transversal.. Esc Anna Nery..

[9] Holcomb DS, Pengetnze Y, Steele A, Karam A, Spong C, Nelson DB. (2021). Geographic barriers to prenatal care access and their
consequences.. Am J Obstet Gynecol MFM..

[10] Tomasi E, Fernandes PAA, Fischer T, Siqueira FCV, Silveira DS, Thumé E (2017). Qualidade da atenção pré-natal na rede básica de saúde do Brasil:
indicadores e desigualdades sociais.. Cad Saude Publica..

[11] Silva EP, Leite AFB, Lima RT, Osório MM. (2019). Prenatal evaluation in primary care in Northeast Brazil: factors
associated with its adequacy.. Rev Saude Publica..

[12] Brasil. (2016). Ministério da Saúde. Protocolos da atenção básica: saúde das
mulheres [Internet].. Brasília: Ministério da Saúde,.

[13] Brito JGE, Santos JMJ, Barreiro MSC, Dantas DDS, Leite AM, Mendes RB. (2021). Participação do companheiro da gestante nas consultas de
pré-natal: prevalência e fatores associados.. Cogitare Enferm..

[14] Moola S, Munn Z, Tufanaru C, Aromataris E, Sears K, Sfetcu R Systematic reviews of etiology and risk.. Aromataris E, Munn Z, editores. JBI manual for evidence synthesis
[Internet]. Adelaide: JBI; 2020. Chapter 7.

[15] Souza PRB, Freitas MPS, Antonaci GA, Szwarcwald CL. (2015). Desenho da amostra da Pesquisa Nacional de Saúde
2013.. Epidemiol Serv Saude..

[16] Stopa SR, Szwarcwald CL, Oliveira MM, Gouvea ECDP, Vieira MLFP, Freitas MPS (2020). Pesquisa Nacional de Saúde 2019: histórico, métodos e
perspectivas.. Epidemiol Serv Saude..

[17] Instituto Brasileiro de Geografia e Estatística. Pesquisa Nacional de Saúde 2019: informações sobre domicílios,
acesso e utilização dos serviços de saúde [Internet].. Rio de Janeiro: IBGE; 2020.

[18] Fundação Oswaldo Cruz. (2024). Pesquisa Nacional de Saúde [Internet].. Rio de Janeiro;.

[19] Brasil, (2012). Ministério da Educação. Resolução nº 466 de dezembro de 2012
[Internet].. Diário Oficial da União; Brasília;.

[20] Ladak Z, Grewal N, Kim MO, Small S, Leber A, Hemani M (2024). Equity in prenatal healthcare services globally: an umbrella
review.. BMC Pregnancy Childbirth..

[21] Krukowski RA, Jacobson LT, John J, Kinser P, Campbell K, Ledoux T (2022). Correlates of early prenatal care access among U.S. women: data
from the Pregnancy Risk Assessment Monitoring System
(PRAMS).. Matern Child Health J..

[22] Raru TB, Mamo Ayana G, Bahiru N, Deressa A, Alemu A, Birhanu A (2022). Quality of antenatal care and associated factors among pregnant
women in East Africa using Demographic and Health Surveys: a multilevel
analysis.. Womens Health..

[23] Denny HM, Laksono AD, Matahari R, Kurniawan B. (2022). The determinants of four or more antenatal care visits among
working women in Indonesia.. Asia Pac J Public Health..

[24] Vasconcelos A, Sousa S, Bandeira N, Alves M, Papoila AL, Pereira F (2022). Antenatal screenings and maternal diagnosis among pregnant women
in Sao Tome & Principe-Missed opportunities to improve neonatal health:
a hospital-based study.. PLOS Glob Public Health..

[25] Arsenault C, Jordan K, Lee D, Dinsa G, Manzi F, Marchant T (2018). Equity in antenatal care quality: an analysis of 91 national
household surveys.. Lancet Glob Health..

[26] Balsells MMD, Oliveira TMF, Bernardo EBR, Aquino PS, Damasceno AKC, Castro RCMB (2018). Avaliação do processo na assistência pré-natal de gestantes com
risco habitual.. Acta Paul Enferm..

[27] Leal MC, Esteves-Pereira AP, Viellas EF, Domingues RMSM, Gama SGN. (2020). Prenatal care in the Brazilian public health
services.. Rev Saude Publica..

[28] Nascimento BTS, Assis JJC, Silva RPD, Lima EM, Barbosa LRDS, Costa KLD (2024). Associação de gravidez na adolescência e
prematuridade.. Brazilian Journal of Implantology and Health Sciences..

[29] Brasil, (2024). Ministério da Saúde, Departamento de Doenças de Condições
Crônicas e Infecções Sexualmente Transmissíveis.. Portal sobre aids, infecções sexualmente transmissíveis, tuberculose e
hepatites virais [Internet]. Brasília;.

[30] Etti M, Lima No AS, Monteiro HS, Araújo MAL, Sousa GS, Castro MC. (2023). Determinants of congenital syphilis in Fortaleza, Brazil: a
retrospective case-control study.. PLOS Glob Public Health..

[31] Motta CT, Moreira MR. (2021). O Brasil cumprirá o ODS 3.1 da Agenda 2030? Uma análise sobre a
mortalidade materna, de 1996 a 2018.. Cien Saude Colet..

