# Supplementary material for: Prenatal tests in Brazil: prevalence and associated factors according to the Brazilian National Health Survey
Source: Rev Esc Enferm USP. 2025 Jan 27;58:e20240154. doi: 10.1590/1980-220X-REEUSP-2024-0154en (PMC11773685; doi:10.1590/1980-220X-REEUSP-2024-0154en)
Supplement: Translation

# Translation: Exames pré-natais no Brasil: prevalência e fatores associados segundo a Pesquisa Nacional de Saúde

**DOI:** 10.1590/1980-220X-REEUSP-2024-0154pt

**Authors:** Francisca Maria da Silva Freitas, Rita Da Graça Carvalhal Frazão Correia, Camila Biazus-Dalcin, Herla Maria Furtado Jorge, Priscila de Souza Aquino, Bruno Luciano Carneiro Alves de Oliveira

**Affiliations:** 1 Universidade Federal do Maranhão, Departamento de Enfermagem, São Luís, MA, Brasil.; 2 University of Dundee, School of Health Sciences, Dundee, Scotland.; 3 Universidade Federal do Piauí, Departamento de Enfermagem, Teresina, PI, Brasil.; 4 Universidade Federal do Ceará, Departamento de Enfermagem, Fortaleza, CE, Brasil.; 5 Universidade Federal do Maranhão, Departamento de Medicina I, São Luís, MA, Brasil.

**Keywords:** Cuidado Pré-Natal, Testes Laboratoriais, Acessibilidade aos Serviços de Saúde, Inquéritos Epidemiológicos, Enfermagem

## Abstract

**Objetivo:**

Analisar a prevalência de exames pré-natais de gestantes e os fatores associados à variação dessa prevalência nos anos da Pesquisa Nacional de Saúde 2013 e 2019.

**Método:**

Estudo transversal, realizado com mulheres que realizaram pré-natal, entrevistadas na Pesquisa Nacional de Saúde 2013 (n = 1.851) e 2019 (n = 2.729).

**Resultados:**

Os exames mais prevalentes foram urina e sangue, e os menos prevalentes foram sífilis e HIV. No período, cresceu a realização dos exames de sífilis (15,2; IC95%: 11,0; 22,0) e HIV (4,3; IC95: 4,3; 8,0), mas reduziu a dos demais. A prevalência de realização dos quatro exames cresceu e alcançou 69,9% (IC95%: 67,0; 72,8) em 2019 ante os 60% (IC95%: 56,1; 63,9) em 2013.

**Conclusão:**

Observou-se maior realização do conjunto dos quatro exames pré-natais, e especificamente para sífilis e HIV, em detrimento da redução na realização dos exames de sangue e urina. Apesar do crescimento no acesso a todos os exames para os grupos e localidades mais vulneráveis do país, ainda é baixa a prevalência nesses grupos.

## INTRODUÇÃO

Os cuidados pré-natais são essenciais à saúde materna e neonatal, pois permitem a identificação precoce e o tratamento oportuno de problemas que possam ocorrer na gestação e pós-parto, além de reduzir a morbimortalidade materna, fetal e infantil^(1)^. No entanto, milhões de gestantes em todo o mundo ainda não os recebem adequadamente. O 3º Objetivo de Desenvolvimento Sustentável (ODS) da Organização das Nações Unidas institui a meta de redução da morbimortalidade materna e infantil, o que requer esforços globais para o alcance do acesso universal aos serviços e insumos de saúde, como o acesso ao pré-natal com realização dos exames^(2,3)^.

Apesar de consistir parte fundamental para o alcance das metas globais, esse acesso ainda representa um problema de grande magnitude^(4)^. Nos Estados Unidos da América (EUA), as disparidades persistem e são significativas. Mulheres nascidas nos EUA apresentam taxas mais elevadas de cuidados pré-natais, comparadas às imigrantes^(5)^. Na Inglaterra, a qualidade do cuidado se mostra insatisfatória pelas desigualdades de acesso e encaminhamento tardio. Ademais, a realização de exames pré-natais ainda não é homogênea entre os grupos populacionais e localidades desse país^(6)^.

Pesquisa com gestantes brasileiras revelou a prevalência de acesso a exames pré-natais inferior a 37,3%^(7)^. Já outra pesquisa realizada com 408 puérperas em três maternidades de referência do estado do Paraná, sul do Brasil, revelou prevalência abaixo de 50% no período gestacional^(8)^. Razões para isso são atribuídas aos Determinantes Sociais de Saúde, na medida em que as características individuais (menor escolaridade, renda, idade, ausência de companheiro) e contextuais (região de moradia e tipo de serviços utilizados) maternas induzem a uma menor realização de exames e de cobertura pré-natal^(9,10,11)^.

O Ministério da Saúde do Brasil^(12)^ preconiza a realização de exames laboratoriais e testes rápidos de rotina durante o pré-natal, incluindo exames de sangue para detecção de sífilis, HIV, hepatites B e C, sorologia para toxoplasmose, diabetes gestacional, anemia e tipagem sanguínea com fator Rh, bem como exames de urina. Esses podem ser realizados na Atenção Primária à Saúde.

Apesar disso, no Brasil, estudos de prevalência e fatores associados à realização de exames pré-natais ainda são menos frequentes na literatura acadêmica do que aqueles sobre a adequação da assistência ao pré-natal a partir da análise do número de consultas e período de início. A ordenação de prioridades do Ministério da Saúde, a organização política em Rede de Atenção à Saúde e a estratégia Rede Cegonha dependem desse conhecimento como forma de avaliar e monitorar a implementação de políticas públicas, organização dos serviços de saúde e manejo do cuidado à gestante e seu recém-nascido^(8,9,10,11,12,13)^.

Dessa forma, análises sobre a prevalência da realização de exames pré-natais e os fatores associados à variação dessa prevalência no Brasil ainda são escassas, o que contribui para manter o desconhecimento sobre os problemas de saúde que acometem as gestantes que envolvem o risco de transmissão vertical na gestação ou trabalho de parto.

Portanto, este estudo buscou analisar a prevalência do acesso a exames pré-natais de gestantes e os fatores associados à variação dessa prevalência no Brasil.

## MÉTODO

### Desenho do Estudo

Trata-se de estudo transversal. A escrita do artigo seguiu as indicações do protocolo *STrengthening the Reporting of OBservational studies in Epidemiology* (STROBE)^(14)^.

### Local

Os dados foram oriundos da Pesquisa Nacional de Saúde (PNS) 2013 e 2019, que é um inquérito domiciliar de base populacional de abrangência nacional. Nela são coletadas informações válidas e representativas da população brasileira sobre suas condições de vida^(15,16,17)^. As bases de dados da PNS foram obtidas no site da FIOCRUZ^(18)^. Todas as etapas de codificação das variáveis e análises foram realizadas pelos autores deste trabalho.

### População e Critérios de Seleção

A população-alvo em 2013 foram os indivíduos com idade ≥18 anos e, em 2019, ≥15 anos, residentes em domicílios particulares permanentes no Brasil. Os questionários abordam os domicílios e todos os seus moradores^(16)^. A amostragem utilizada foi probabilística por conglomerado em três estágios de seleção, com estratificação pelas áreas selecionadas^(17)^. Maiores detalhes podem ser obtidos em publicações da PNS^(15,17)^.

### Definição da Amostra

Neste estudo, foram consideradas apenas mulheres de 18 a 49 anos de idade que estiveram grávidas nos dois anos anteriores à data da coleta dos dados da PNS e fizeram pré-natal. Em 2013, 1.851 mulheres compuseram a amostra, e, em 2019, outras 2.729.

### Análise e Tratamento dos Dados

Na PNS, as mulheres que estiveram grávidas nos dois anos antes da data da entrevista foram questionadas sobre a realização de exame de sangue (sem considerar o teste de gravidez), exame de urina (sem considerar o teste de gravidez), exame de sífilis e HIV/AIDS, e local de realização do pré-natal (somente público, somente privado, e público e privado). Para esses exames ou testes, as respostas possíveis foram “sim”, “não”, “não sabe/não lembra” ou “ignorado”. Para cada um deles, as respostas foram agregadas: sim vs as demais. Os quatro exames também foram agregados para se verificar a realização conjunta de todos eles (sim, não). Para ambos os anos da PNS, foram estimadas as prevalências e seus Intervalos de Confiança de 95% (IC95%) das variáveis socioeconômicas, demográficas e local do pré-natal. Diferenças na distribuição das frequências das variáveis foram verificadas segundo o ano, consideradas estatisticamente significante ao nível de 5% na ausência de sobreposição dos IC95%. Teste qui-quadrado de Pearson foi utilizado para confirmar diferenças entre as duas PNS.

A prevalência e os respectivos IC95% dos exames individuais e em conjunto foram estimados para cada ano da PNS. A mudança da prevalência nos dois anos da PNS foi apresentada por meio da diferença absoluta. A magnitude dessa variação no período foi computada com Modelo Linear Generalizado (GLM), usando a distribuição Gaussiana. Para realização da análise dos dados no presente estudo, os dados dos dois inquéritos foram agregados em um único banco. Usando-se o peso do morador selecionado com calibração, foram calculadas as mudanças absolutas de 2013 a 2019 segundo características sociodemográficas, de paridade e locais do pré-natal. Para calcular a mudança de prevalência de 2013 para 2019, foi modelado o efeito do ano sobre o desfecho segundo a variável de agrupamento. A taxa de prevalência percentual relatada foi calculada como o exponencial do coeficiente menos um e multiplicado por 100. Por fim, foram realizados modelos de regressão de Poisson, variância robusta, bruta e ajustada por variáveis sociodemográficas, de paridade e local do pré-natal para testar a associação do ano da PNS (2019 vs 2013) com o acesso a exames pré-natais. Todas as análises foram feitas no *software* RStudio, versão 2023.6.1.524 (*R Foundation for Statistical Computing*, Boston, *United States of America*).

### Aspectos Éticos

Os dados da PNS 2013 e 2019 são de domínio público, e podem ser utilizados de acordo com as pesquisas de interesse. As pesquisas foram aprovadas pela Comissão Nacional de Ética em Pesquisa/Conselho Nacional de Saúde (Processo nº 328.159, emitido em julho de 2013; Processo nº 3.529.376, emitido em agosto de 2019). Todas as entrevistadas assinaram o Termo de Consentimento Livre e Esclarecido^(19)^.

## RESULTADOS

A amostra foi composta a partir de dados de 4.580 gestantes entrevistadas. Os dados relativos às características sociodemográficas, de paridade e local do pré-natal das gestantes na PNS 2013 e 2019 foram dispostos na Tabela 1.

**Tabela 1:** Características sociodemográficas, de paridade e local do pré-natal de gestantes entrevistadas na Pesquisa Nacional de Saúde – Brasil, 2013 e 2019.

Características	2013 (n = 1.851)			2019 (n = 2.729)			p-valor^1^
	n	%	IC95%	n	%	IC95%	
**Total**	1801	97,3	96,4; 98,3	2680	98,2	97,6; 98,9	
Faixa etária (em anos)							
18 a 29	1135	61,3	57,5; 65,0	1441	52,8	49,5; 56,0	***0,001***
30 a 39	652	35,2	31,6; 38,3	1133	41,5	38,9; 44,8
40 a 49	65	3,5	2,2; 4,3	156	5,7	4,7; 7,2
Cor/raça							
Parda	926	50,0	46,2; 53,8	1397	51,2	48,0; 54,5	
Branca	739	39,9	36,1; 43,7	980	35,9	32,8; 39,1	0,20
Preta	163	8,8	6,7; 10,9	308	11,3	9,2; 13,5
Outras	24	1,3	0,6; 2,0	41	1,5	1,0; 2,1
Estado civil							
Sem cônjuge	1048	56,6	52,9; 60,4	1586	58,1	55,0; 61,2	
Com cônjuge	803	43,4	39,6; 47,1	1143	41,9	38,8; 45,0	0,56
Paridade							
Um parto	731	39,5	35,8; 43,3	1064	39	36,1; 42,0	
Dois a quatro partos	1024	55,3	51,5; 59,2	1536	56,3	53,3; 59,3	0,86
Cinco ou mais partos	94	5,1	3,9; 6,4	128	4,7	3,5; 5,9	
Participação em atividades religiosas							
Sim	857	46,3	42,5; 50,1	1318	48,3	45,0; 51,5	0,45
Não	994	53,7	49,9; 57,5	1411	51,7	48,5; 55,0	
Plano de saúde							
Sim	515	27,8	24,0; 31,6	742	27,2	24,0; 30,3	0,82
Não	1336	72,2	68,4; 76,0	1987	72,8	69,7; 76,0	
Escolaridade							
Até fundamental incompleto ou equivalente	426	23,0	19,9; 26,0	491	18,0	15,7; 20,2	***0,001***
Médio incompleto ou equivalente	446	24,1	20,8; 27,3	519	19,0	16,5; 21,5	
Superior incompleto ou equivalente	761	41,1	37,1; 45,0	1190	43,6	40,4; 46,8	
Superior completo	220	11,9	9,5; 14,3	532	19,5	16,7; 22,2	
Renda domiciliar *per capita*							
Até ½ salário mínimo	796	43,0	39,1; 46,8	1214	44,5	41,4; 47,7	0,95
½ até 1 salário mínimo	546	29,5	26,2; 32,9	780	28,6	25,4; 31,7	
1 até 2 salários mínimos	324	17,5	14,2; 20,8	445	16,3	13,6; 19,1	
2 até 3 salários mínimos	76	4,1	2,5; 5,6	117	4,3	2,8; 5,9	
Mais de 3 salários mínimos	109	5,9	4,2; 7,6	169	6,2	4,8; 7,7	
Macrorregião do país							
Sudeste	707	38,2	34,3; 42,2	1051	38,5	35,4; 41,6	
Nordeste	533	28,8	25,5; 32,1	778	28,5	26,2; 30,7	
Sul	270	14,6	11,9; 17,4	355	13,0	11,5; 14,6	0,81
Norte	185	10,0	8,5; 11,6	308	11,3	10,1; 12,6	
Centro-Oeste	154	8,3	7,0; 9,6	237	8,7	7,4; 9,9
Tipo de cidade							
Interior	1068	57,7	54,2; 61,2	1676	61,4	58,8; 64,0	0,18
Capital ou Região Metropolitana	783	42,3	38,8; 45,8	1053	38,6	36,0; 41,2		
Local em que fez o pré-natal							
Somente público	1283	69,3	65,5; 73,0	1771	64,9	61,6; 68,2	0,06
Somente privado	500	27,0	23,3; 30,6	794	29,1	26,0; 32,2	
Público e privado	70	3,8	2,5; 5,1	164	6,0	4,7; 7,3	

Nota: IC95% – Intervalo de Confiança de 95%

^1^teste qui-quadrado de Pearson.

Fonte: PNS 2013 e 2019.

A proporção de gestantes que declarou ter realizado pré-natal no Brasil aumentou 0,9% nos anos. Apesar de a faixa etária de 18 a 29 anos ser a mais prevalente, foram observados redução nesse intervalo e aumento das demais faixas etárias. Predominaram as gestantes pardas, sem cônjuge, com dois a quatro partos, sem atividades religiosas, sem plano de saúde, com renda de até ½ salário mínimo, residentes na região Sudeste, no interior e que realizaram o pré-natal somente em serviços públicos. Observaram-se, nos anos, crescimento significativo de gestantes com maior escolaridade, superior incompleto e superior completo, com consequente redução das menores escolaridades. Os dados referentes à prevalência dos exames realizados pelas gestantes estão dispostos na Tabela 2.

**Tabela 2:** Prevalência e mudança da prevalência da realização de exames pré-natais de gestantes entrevistadas na Pesquisa Nacional de Saúde – Brasil, 2013 e 2019.

Exames	2013 (n = 1.851)			2019 (n = 2.729)			Mudança absoluta* (2019 vs 2013)		
	n	%	IC95%	n	%	IC95%	%	IC95%
Urina	1816	98,1	97,2; 98,9	2541	93,1	91,2; 95,0	–5,0	–6,8; –2,8
Sangue	1801	97,3	96,2; 98,4	2573	94,3	92,6; 96,1	–3,0	–4,9; –0,9
HIV	1575	85,1	82,3; 87,8	2440	89,4	87,9; 91,1	4,3	1,0; 8,0
Sífilis	1196	64,6	60,8; 68,5	2178	79,8	77,4; 82,2	15,2	11,0; 22,0
**Total**	1111	60,0	56,1; 63,9	1908	69,9	67,0; 72,8	9,9	5,0; 16,0

Nota: IC95% – Intervalo de Confiança de 95%

*GLM.

Fonte: PNS 2013 e 2019.

Conforme observa-se na Tabela 2, houve redução significativa na realização de exames de sangue e urina pelas gestantes entrevistadas e aumento significativo na realização de exames de sífilis, HIV e no total de exames nos anos da PNS. Os dados referentes à realização do conjunto de exames pré-natais pelas Unidades de Federação foram dispostos na Figura 1.

**Figura 1:** Prevalência (%) e Intervalos de Confiança de 95% da realização do conjunto de exames pré-natais de gestantes entrevistadas na Pesquisa Nacional de Saúde, Brasil, 2013 e 2019.

Fonte: Brasil, PNS 2013 e 2019.

Notas: Entre os anos, todos os exames p-valor < 0,01.

Observa-se na Figura 1 em 2013 que houve diferença significativa no acesso ao pré-natal: o Ceará apresentou a menor prevalência quando comparado ao Amapá, Rondônia, Roraima, Tocantins, Goiás, Mato Grosso do Sul e Paraná. Já em 2019, as menores estimativas foram dos estados Maranhão, Ceará e Minas Gerais. Houve uma diferença significativa entre Maranhão, que apresentou a menor prevalência, e Acre, Amapá e São Paulo. Nos anos, somente o estado do Acre apresentou aumento estatisticamente significativo, bem como o Brasil de modo geral, denotando aumento do acesso à realização dos exames investigados.

Os dados referentes à realização dos exames pré-natais segundo características sociodemográficas, de paridade e local de realização do pré-natal das gestantes foram dispostos na Tabela 3.

**Tabela 3:** Prevalência e mudança da prevalência da realização de exames pré-natais segundo características sociodemográficas, de paridade e local de realização do pré-natal de gestantes entrevistadas na Pesquisa Nacional de Saúde – Brasil, 2013 e 2019.

Características	2013 (n = 1.851)		2019 (n = 2.729)		Mudança absoluta* (2019 vs 2013)	
	%	IC95%	%	IC95%	%	IC95%
**Total**	60,0	56,1; 63,9	69,9	67,0; 72,8	9,9	5,0; 16,0
Faixa etária (em anos)						
18 a 29	55,1	49,9; 60,3	67,1	63,0; 71,2	12,1	5,0; 21,0
30 a 39	67,7	61,7; 73,8	73,3	69,0; 77,5	6,0	–1,6; 14,0
40 a 49	67,5	52,7; 82,3	71,2	59,6; 82,8	3,7	–13,9; 25,0
Cor/raça						
Branca	62,5	55,9; 69,1	68,5	63,1; 74,0	6,0	–2,4; 16,0
Parda	58,2	53,0; 63,4	71,6	68,0; 75,1	14,0	7,0; 22,0
Preta	58,2	45,5; 71,0	68,7	60,5; 76,8	10,5	–4,4; 29,0
Outras	62,2	34,3; 90,2	55,9	37,7; 74,1	–6,3	–32,2; 30,0
Estado civil						
Com cônjuge	62,9	57,1; 68,8	70,6	66,2; 75,0	8,0	1,0; 16,0
Sem cônjuge	57,7	52,6; 62,9	69,4	65,5; 73,2	11,7	5,0; 20,0
Paridade						
Um parto	65,0	58,1; 70,9	69,2	64,2; 74,1	5,0	–3,2; 13,0
Dois a quatro partos	56,8	51,5; 62,0	70,7	67,1; 74,4	15,0	8,0; 22,0
Cinco ou mais partos	60,1	48,5; 71,7	66,0	53,1; 79,0	6,0	–10,9; 26,0
Participa de atividades religiosas						
Sim	62,4	56,4; 67,9	72,2	68,4; 76,4	9,8	3,0; 18,0
Não	58,0	53,0; 62,9	67,8	63,8; 71,8	9,8	3,0; 18,0
Plano de saúde						
Sim	69,2	61,5; 76,8	75,7	70,0; 81,4	6,5	–2,9; 17,0
Não	56,5	52,0; 61,0	67,7	64,4; 71,1	11,2	6,0; 18,0
Escolaridade						
Até fundamental incompleto ou equivalente	42,8	35,8; 49,9	57,8	51,2; 64,4	15,0	6,0; 27,0
Médio incompleto ou equivalente	60,0	52,4; 67,7	64,9	58,0; 71,7	4,9	–5,0; 16,0
Superior incompleto ou equivalente	65,8	59,1; 72,4	74,1	69,8; 78,4	9,0	–1,0; 17,0
Superior completo	73,0	62,8; 83,2	76,6	70,4; 82,9	4,0	–8,2; 17,0
Renda domiciliar *per capita*						
Até ½ salário mínimo	51,0	45,4; 56,7	66,4	62,4; 70,5	17,0	9,0; 25,0
½ até 1 salário mínimo	66,6	59,9; 73,4	70,2	64,3; 76,1	4,0	–5,4; 14,0
1 até 2 salários mínimos	63,4	52,0; 74,8	79,0	72,1; 85,9	15,6	–2,0; 34,0
2 até 3 salários mínimos	62,7	42,7; 82,8	70,5	57,2; 83,8	7,8	–15,0; 37,0
Mais de 3 salários mínimos	79,8	68,0; 91,6	69,0	57,7; 80,4	–10,2	–23,4; 5,0
Macrorregião do país						
Norte	63,8	56,1; 71,6	71,5	66,3; 76,8	7,7	–1,2; 19,0
Nordeste	51,5	44,9; 58,1	67,2	62,7; 71,7	17,0	8,0; 27,0
Centro-Oeste	72,0	64,6; 79,4	70,1	63,3; 76,9	–1,2	–11,3; 9,0
Sudeste	60,1	52,4; 67,7	70,6	64,6; 76,5	10,5	–1,0; 22,0
Sul	67,1	57,2; 76,9	72,3	65,9; 78,8	5,0	–6,5; 19,0
Tipo de cidade						
Capital ou Região Metropolitana	68,2	63,8; 72,5	73,0	69,4; 76,7	4,8	–0,9; 11,0
Interior	54,0	48,1; 60,0	67,9	63,8; 72,0	15,0	7,0; 24,0
Local em que fez o pré-natal						
Somente privado	73,0	66,3; 79,7	72,2	66,5; 77,8	–0,8	–8,9; 8,0
Somente público	54,5	49,8; 59,2	67,5	63,9; 71,0	14,0	7,0; 21,0
Público e privado	72,4	66,0; 78,8	74,4	69,4; 79,3	2,0	–5,8; 1,0

Notas: IC95% – Intervalo de Confiança de 95%

*GLM.

Fonte: PNS 2013 e 2019.

Conforme observa-se na Tabela 3, a realização dos exames aumentou nos anos das pesquisas, com diferença estatisticamente significante para as gestantes mais jovens (18 a 29 anos), pardas, sem cônjuge, com dois a quatro partos, sem plano de saúde, de menor escolaridade, menor renda, residentes na região Nordeste, no interior e com pré-natal realizado somente na rede pública.

A Figura 2 evidencia a associação bruta e ajustada do ano da PNS com a realização simultânea e específica dos exames pré-natais.

**Figura 2:** Associação bruta e ajustada de exames pré-natais de gestantes com o ano em que foram entrevistadas na Pesquisa Nacional de Saúde, Brasil, 2013 e 2019.

Fonte: Brasil, PNS 2013 e 2019.

*Regressão de Poisson com variância robusta. Ajuste realizado em função das variáveis presentes na Tabela 1.

Conforme observa-se na Figura 2, verificou-se melhoria do acesso simultâneo a todos os exames. Em 2019, em relação ao ano de 2013, após o ajuste pelos fatores de confusão, gestantes tiveram chance 13% (RP = 1,13; IC95%: 1,07–1,20) maior de realização dos exames investigados. Ao se detalhar por exame, análise ajustada também indicou melhora no acesso à realização de exames de sífilis e HIV, com maiores chances: 21% (RP = 1,21; IC95%: 1,15–1,27) e 4% (RP = 1,04; IC95%: 1,03–1,06), respectivamente. Por outro lado, gestantes apresentaram redução no acesso à realização dos exames de sangue (RP = 0,97; IC95%: 0,96–0,98) e urina (RP = 0,95; IC95%: 0,94–0,96).

## DISCUSSÃO

Os resultados deste estudo apontaram aumento na realização do conjunto dos quatro exames pré-natais, especialmente impulsionado pelos exames de sífilis e HIV no período de 2013 a 2019 no Brasil. Embora seja elevada a prevalência, houve redução do acesso aos exames de sangue e urina. O avanço ao conjunto dos exames observado foi para os grupos e localidades mais vulneráveis do país. Contudo, ainda persistem desigualdades no acesso, em âmbitos individuais e contextuais, evidenciando a necessidade de intensificar esforços para acesso mais equânime aos exames laboratoriais pré-natal em todo o Brasil. Está comprovado que o acesso oportuno, apropriado e equitativo a cuidados de saúde de qualidade durante a gravidez contribui para melhores resultados de saúde das mulheres que dão à luz e dos bebês após o nascimento^(20)^.

Revisão abrangente realizada com 68 revisões sistemáticas de 20 países diferentes sobre equidade dos serviços de saúde evidenciou algumas barreiras para o acesso pré-natal, como pacientes não brancas, com menor escolaridade, menor renda e residentes em áreas de localização remota^(20)^.

No presente estudo, no comparativo entre os estudos das PNS de 2013 e 2019, houve um avanço significativo da realização dos exames para o grupo de gestantes entre a menor faixa de idade do estudo (18 a 29 anos) e de menor escolaridade. Nos EUA, mulheres mais jovens, com menos de 24 anos e com menor escolaridade confirmaram mais tardiamente a gravidez e receberam cuidados pré-natais mais tardiamente do que desejavam, o que pôde dificultar a adequada assistência e realização dos exames^(21)^. Outro estudo realizado em 19 países da África Oriental sobre a qualidade dos cuidados pré-natais e fatores associados entre mulheres grávidas apontou semelhança sobre esses resultados, ao revelar que a qualidade desse cuidado foi maior na faixa de idade dos 20 aos 34 anos (69,44%) e com maior escolaridade^(22)^. Já estudo realizado na Indonésia com 8.239 mulheres trabalhadoras evidenciou maior acesso às consultas pré-natais naquelas com parceiros, com maior nível educacional, menor paridade, maiores rendas e plano de saúde, o que coaduna com os dados do presente estudo^(23)^.

Na República Democrática de São Tomé e Príncipe, das gestantes que tiveram o mínimo de oito consultas, apenas 34% conseguiram realizar dois exames de hemoglobina e dois de urina, evidenciando que, mesmo com o número de acompanhamento adequado, as gestantes não conseguiram realizar o rastreio recomendado durante a gestação. Considerando a realização de pelo menos um exame, 95,5% tiveram rastreamento para HIV, 91,2%, para sífilis, 60,1%, para hepatite, 75,5%, para hemoglobina, 54,6%, para glicemia, e 70,3%, para urina. A justificativa para a não realização foram a ausência de reagente e a falta de apoio econômico^(24)^. Em outro estudo em Lusaka, na Zâmbia, sobre os fatores associados ao rastreio do HIV e da sífilis entre grávidas na primeira consulta pré-natal, apenas 29% delas fizeram o rastreio da sífilis, em comparação com 95%, que fizeram o rastreio do HIV na primeira consulta pré-natal^(25)^.

Estudo realizado em Fortaleza, região Nordeste do Brasil, ao analisar 560 consultas pré-natais, evidenciou que apenas 25,4% das gestantes realizaram todos os exames preconizados adequadamente. Essa foi a menor adequabilidade encontrada na assistência pré-natal, em detrimento da realização dos procedimentos clínico-obstétricos e número de consultas e acesso precoce. As prevalências de adequabilidade foram 30,9% para HIV, 36,2% para sífilis e 34,4% para urina^(26)^.

Um achado que merece atenção é a melhora de acesso para os grupos de maior vulnerabilidade. No entanto, o que ainda se observa é a marcante desigualdade no acesso e na qualidade do cuidado pré-natal com sérias repercussões maternas e infantis relacionadas, em grande parte, às dificuldades geográficas, barreiras de acesso a serviços diagnósticos e terapêuticos^(27)^. Isto revela a necessidade urgente de melhorias da adequação pré-natal para um cuidado integral que responda às demandas relacionadas às vulnerabilidades sociais e econômicas, sendo primordial para a prevenção e detecção precoce de síndromes e patologias maternas e fetais por meio dos exames laboratoriais e, consequentemente, da redução das mortes^(28)^.

Deve-se destacar que, desde 2016, o Ministério da Saúde declarou epidemia de sífilis no Brasil. Contudo, apesar dos esforços para aumentar a testagem e o tratamento da infecção entre as mulheres grávidas, as taxas de sífilis congênita no país permanecem elevadas^(29)^. Evidencia-se que a relação da sífilis congênita está diretamente associada a fatores de vulnerabilidade socioeconômica. Estes determinantes não afetam apenas o acesso a serviços essenciais de cuidados pré-natais, mas também a continuidade e a qualidade de tais medidas preventivas^(30)^. Ademais, ressalta-se que o conhecimento acerca do exame de sífilis do parceiro é primordial para interrupção da cadeia de transmissão, porém esse dado é difícil de ser analisado, uma vez que em 2019 (21,9%) e em 2013 (99,6%) foram registrados “não se aplica”, com mulheres sem parceiro.

Existem grandes desafios para a melhoria da oferta do cuidado pré-natal e acesso aos seus exames. Percebe-se a urgente necessidade de políticas de saúde que contribuam com o acesso a exames laboratoriais pré-natais na população vulnerável, para que, assim, consiga-se reduzir as desigualdades em saúde e seus efeitos nos indicadores de mortalidade materno-infantil no país. Esta ação faz parte das metas do ODS até 2030, e, para isso, requer a qualificação desses serviços^(31)^.

### Limitações e Contribuições do Estudo

Apesar desses resultados, há algumas limitações. Dados de inquéritos epidemiológicos em que as gestantes autorreferiram as informações dos exames podem apresentar viés recordatório e de informação. Houve mudanças substanciais nas PNS em relação à forma que se realizou a pergunta sobre os exames (em 2013: durante o pré-natal, a Sra. fez exame de sangue? E em 2019: nesta gravidez, você fez algum exame de sangue, sem considerar o teste de gravidez?). A abordagem distinta para o mesmo exame pode ter influenciado o entendimento e a informação processados durante a entrevista, interferindo nas respostas das gestantes. Outro ponto importante está relacionado à não especificação de demais exames, além de HIV e sífilis, e à impossibilidade de distinção do tipo de exame de sangue e urina realizados (se glicemia de jejum, hemograma completo, sorologias).

Contudo, trata-se de inquérito nacional com gestantes de todo o Brasil. Os resultados indicam a importância de se investigar o acesso aos exames de rotina pré-natal. Pode contribuir para reformulação de estratégias que melhorem a qualidade do pré-natal no país, além de contribuir para o conhecimento dos profissionais de saúde para a tomada de decisão assertiva para um cuidado às gestantes, tendo em vista o perfil epidemiológico de vulnerabilidade apresentado.

## CONCLUSÃO

Este estudo apontou aumento na realização do conjunto dos quatro exames pré-natais e também especificamente para os exames de sífilis e HIV no período de 2013 a 2019 no Brasil. Porém, houve redução dos exames de sangue e urina. Este avanço foi para os grupos e localidades mais vulneráveis do país, tais como gestantes mais jovens (18 a 29 anos), pardas, sem cônjuge, com dois a quatro partos, sem plano de saúde, de menor escolaridade, menor renda, residentes na região Nordeste, no interior e com pré-natal realizado somente na rede pública. No entanto, ainda é baixa a prevalência, observando-se ainda iniquidades de acesso à realização dos exames no Brasil.

Apesar da importância dessa temática, estudos de âmbito nacional publicados ainda não realizam uma análise dos exames pré-natais preconizados no país. Assim, este estudo indica um caminho para uma pauta de pesquisa sobre a importância da investigação do acesso aos exames pré-natais, podendo contribuir para reformulação de estratégias de intervenção que visem à melhoria dos indicadores de assistência na população gestante usuária do Sistema Único de Saúde.

Além disso, vem contribuir com a produção do conhecimento na área obstétrica no âmbito da Atenção Primária à Saúde, favorecendo maior direcionamento de ações às gestantes vulneráveis, bem como auxiliando na avaliação da realidade brasileira, favorecendo a comparação com os demais países.
